# Evaluation of patients’ knowledge about oral anticoagulant medicines and use of alert cards by community pharmacists

**DOI:** 10.1007/s11096-020-01134-w

**Published:** 2020-09-07

**Authors:** Yogini H. Jani, Bindiya Hirani, Carina Livingstone

**Affiliations:** 1 Centre for Medicines Optimisation Research and Education, University College London Hospitals NHS Foundation Trust, 235 Euston Road, London, NW1 2BU UK; 2 UCL School of Pharmacy, 29-39 Brunswick Square, London, WC1N 1AX UK; 3 NHS Specialist Pharmacy Service, Medicines Use and Safety, Ground Floor, The Causeway, Worthing, West Sussex BN12 6BT UK

**Keywords:** Anticoagulant alert card, Community pharmacy practice, Oral anticoagulant, Patient knowledge, Patient safety

## Abstract

*Background* Anticoagulants continue to pose high risk of harm to patients despite the discovery of novel direct-acting oral anticoagulant agents that require less monitoring than warfarin. *Objective* To evaluate patients’ knowledge about their oral anticoagulants and the potential role for community pharmacists in optimising safety. *Setting* Community pharmacies in England. *Methods* An online survey-based evaluation conducted over a 5-month period to ascertain patients’ knowledge, use of anticoagulant alert cards, compliance with national monitoring requirements for warfarin, and frequency and nature of community pharmacist involvement in optimisation. Differences between patients on direct-acting oral anticoagulant agents and warfarin were assessed using Chi squared tests. *Main outcome measure* Patients’ knowledge and use of anticoagulant alert cards. *Results* A total of 1515 pharmacies participated. Of 22,624 patients, 97% knew that they were taking anticoagulants; 20% had alert cards with them at time of dispensing; 17% had no card and 10% refuted their usefulness. Patients on warfarin were more aware of interactions with over-the-counter or herbal medicines than those on direct-acting oral anticoagulant agents. Of the patients on warfarin, 82% confirmed monitoring in the previous 12 weeks in accordance with national standards, with the international normalised ratio value known for 76%. Pharmacists intervened in a fifth of the patients to issue an alert card, contact the general practitioner for a change in the prescription or due to interacting medicines. *Conclusion* Patients had reasonable knowledge of their anticoagulation therapy, but areas for improvement were identified. Community pharmacists are well placed to optimise the safe use of anticoagulants.

## Impacts on practice

- Few patients carry anticoagulant alert cards; pharmacists should provide these at point of dispensing to help address knowledge gaps and identify safety concerns.
- Information provision is particularly important for patients on direct-acting oral anticoagulant agents, who may be less aware of the nature of and reason for their anticoagulant treatment.
- Pharmacists should work in partnership with patients and prescribers to increase knowledge and awareness of potential interactions of anticoagulants with other medicines.

## Introduction

Use of anticoagulants has increased over the years with an overall 134.7% increase in the number of anticoagulant prescriptions dispensed between 2006 and 2016 in England [1]. Anticoagulants are high risk medicines [2, 3]; they have a heightened risk of causing significant harm when used in error and are frequently identified as a cause of preventable harm and admission to hospital. Warfarin, a vitamin K antagonist (VKA), is most commonly used and is widely known to health professionals, but newer direct-acting oral anticoagulants (DOACs) may not be recognised as anticoagulants and this has contributed to various medication errors [4]. Reported errors include inappropriate continuation or discontinuation of anticoagulant therapy, inadvertent co-prescribing with interacting drugs, which increase the risk of bleeding (such as non-steroidal anti-inflammatory drugs (NSAIDs) and antiplatelet agents), and concomitant prescribing with other anticoagulants (such as heparin or warfarin) [4].

Patient education and improved understanding of their anticoagulant therapy has been shown to improve patient safety and help avoid preventable anticoagulant related adverse effects [5, 6]. The National Patient Safety Agency in England (now NHS Improvement) issued a patient safety alert with actions to improve anticoagulant safety, which included providing specific patient information, regular blood monitoring and checking drug interactions and subsequently updated this to include DOACs and advised that patients on anticoagulant therapy should always carry a standard yellow (credit card sized) alert card to inform health and social care practitioners that they are taking an anticoagulant.

The National Institute for Health and Care Excellence (NICE) has highlighted that information and awareness are essential in ensuring safe and effective use of anticoagulants [4].

A fundamental issue with all patient held information is patient understanding and uptake. Many studies report poor utilisation of patient held medical records by patients themselves [7]. They are too often made to be passive recipients of medicines and not informed and empowered to play their part in making the process of medication safer [8]. There is evidence of the role of community pharmacists in improving safety and adherence through monitoring and education [9, 10], identifying and mitigating interactions with over the counter medicines [11] and better management of anticoagulant therapy through collaboration between the pharmacist and the GP [12, 13].

Patient and public involvement is recommended as one of the four target areas identified by the World Health Organisation Global Patient Safety Challenge, ‘Medication Without Harm’ [8]. Our experience from two previous community pharmacy medicines safety audits made available across England is that collaborative audit-based evaluations can provide a successful large-scale means of raising awareness of key safety issues and drive improvement [14, 15].

## Aim of the study

The primary aim of the study was to evaluate patient awareness of key knowledge about their anticoagulant medicine and the use of patient held anticoagulation alert cards. Secondary aims were to assess compliance with national alert recommendations including monitoring requirements for warfarin and explore the role of community pharmacists in anticoagulant safety.

## Ethics approval

According to the NHS Health Research Authority decision tool and guidance [16], this study was a service evaluation as it was “designed and conducted solely to define or judge current care” and therefore did not require NHS research ethics approval.

## Methods

Community pharmacies in England were invited to participate in the evaluation as part of their contractual requirements under NHS regulations, which requires participation in two clinical audits per year [17]. Information about the study was published on the NHS Specialist Pharmacy Service (SPS) and Pharmaceutical Services Negotiating committee (PSNC) websites in November 2017 and circulated to multiple pharmacy networks including the Community Pharmacy Patient Safety Group, Local Pharmaceutical Committees, Medicines Use and Safety (MUS) Networks, Local Professional Network Leads and Chief Pharmacist Groups.

All patients who presented a prescription for an oral anticoagulant, whether VKA (acenocoumarol, phenindione, and warfarin) or DOAC (apixaban, dabigatran, edoxaban and rivaroxaban) at participating community pharmacies were eligible. A minimum sample of 15 patients or 2 weeks per community pharmacy was recommended.

An online survey-based tool was developed by a stakeholder group led by SPS-MUS and included representatives from NHS Improvement, Day Lewis Pharmacy, Pinnacle Health Partnership, PSNC, the Royal Pharmaceutical Society and the Community Pharmacy Patient Safety Group. The survey was based on the audit checklist for the national patient safety alert [18] and consisted of 29 questions addressing four areas of interest: patient demographics and concomitant medicines; patient knowledge; use of patient held alert cards; assessment of compliance with national guidance for safety indicators and monitoring of patients on warfarin; and the final section for any general comments including actions taken by the community pharmacist in response to a safety issue. Key knowledge was defined, on the basis of the national alert [18], as patients being aware that they are taking an anticoagulant medicine i.e. a medicine to thin the blood/prevent blood clots, and that are aware they need to check with a doctor or pharmacist before taking over-the-counter medicines or herbal products. A mixture of open and closed questions were used, with one five-point Likert scale to assess usefulness of alert cards, and the option to add free text comments. The tool was revised and finalised following pilot testing in 10 community pharmacies from the Day Lewis group [19]. Data was reported via PharmOutcomes, a secure platform used by pharmacies in England.

### Data analysis

Responses were extracted from the centralised database, collated in Microsoft Excel^®^ and transferred to Statistical Package for Social Sciences (SPSS) program version 25 for analysis. Data was cleansed to aid analysis; responses where the age was entered as more than 100 or if anticoagulant name was missing were excluded; individual survey items with missing items were included for analysis.

Descriptive statistics were used for patient demographics, medicines prescribed, possession of alert card, knowledge and awareness of anticoagulation medicine; Chi squared test of independence was used to assess the relationship between knowledge and awareness of anticoagulation medicine and factors that may influence this, such as type of anticoagulant (DOAC or VKA), domiciliary status, use of medicine compliance aid, and the possession of alert card. For patients on warfarin, percentage compliance with the national recommended standard for monitoring of international normalised ratio (INR) within the previous 12 weeks was assessed. Free text responses were analysed using an inductive thematic approach.

## Results

**Fig. 1:**
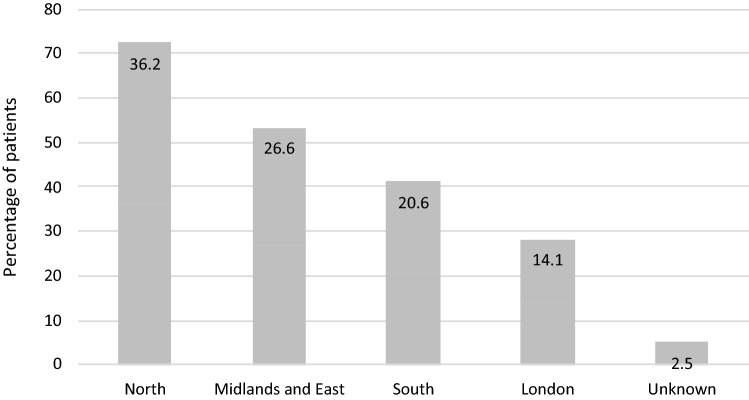
The geographic distribution of audit responses

1515 pharmacists participated and submitted data for 22,684 patients over a 5-month period between November 2017 and March 2018. 22,624 cases were included in the analysis; 42 patients with an age less than 18, 9 patients with an age entered as over 100 and 9 patients where the name of the anticoagulant was not stated were excluded. Most of the patients were from London (14.1%) or Yorkshire and Northern regions (12.5%) as shown in Fig. 1. The median age of patients was 75 years (range 18–100 years) with more male patients (57.6%) compared to female patients (42.4%). Warfarin was the most commonly prescribed oral anticoagulant (47.7%). Table 1 provides further details of gender, domiciliary status, oral anticoagulant prescribed and use of a multi-compartment compliance aid (MCA) by patients.

**Table 1:** Patient demographics

Total = 22,624	Number of patients (percentage)
Male; mean age in years (standard deviation)	13,035 (57.6%); 72.19 (± 12.2)
Female; mean age in years (standard deviation)	9589 (42.4%); 74.7 (± 12.9)
Care home resident	517 (2.3%)
Not care home resident	21,650 (95.7%)
Residential status unknown	457 (2%)
Multi-compartment compliance aid used	2420 (10.7%)
*Anticoagulant prescribed*	
Acenocoumarol	75 (0.3%)
Apixaban	5493 (24.3%)
Dabigatran	728 (3.2%)
Edoxaban	281 (1.2%)
Phenindione	2 (0.01%)
Rivaroxaban	5243 (23.2%)
Warfarin	10,802 (47.7%)

*Antiplatelet agent co-prescribed*	*Total = 1340 (5.9%)*
Aspirin	898 (4%)
Clopidogrel	400 (1.8%)
Dipyridamole	6 (0.04%)
Ticragrelor	2
Aspirin and clopidogrel	31 (0.13%)
Aspirin and ticragrelor	1
*Method of data collection (*^a^ *patient not contacted)*	
Conversation with the patient in the pharmacy	12,944 (57.2%)
Conversation with the patient by telephone	4046 (17.9%)
Patient’s representative in pharmacy^a^	2308 (10.2%)
Medicine delivered by pharmacy^a^	3326 (14.7%)

### Concomitant drugs

Seventeen patients were reported with multiple anticoagulants prescribed. In 15 cases, warfarin was prescribed in combination with another anticoagulant. 1340 (6%) of patients were also on at least one antiplatelet agent. One-third of all patients were taking a proton-pump inhibitor or H_2_-antagonist; with the proportion of patients on DOACs being higher than those on VKA (37.8%, 4436/11,749 vs. 31.5%, 3434/10,884; *p* < 0.001, Chi squared test) (Table 1).

### Patient knowledge, awareness and use of alert cards

**Fig. 2:**
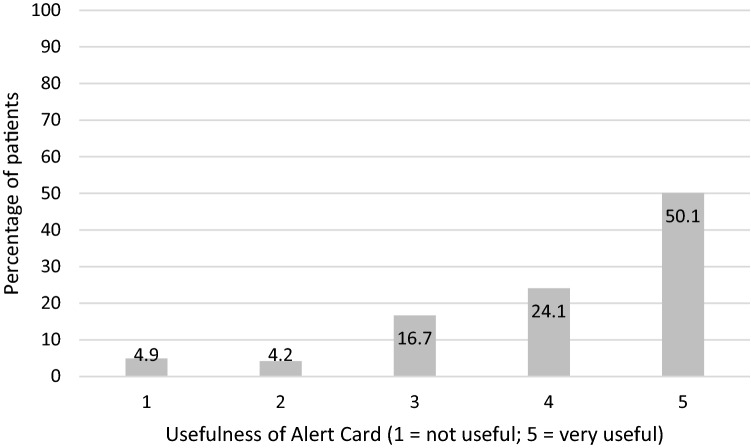
Patients perceived usefulness of alert cards

A total of 16,994 (75%) of patients were contacted directly in person or by phone. Of these, 14,274 (84%) patients had key knowledge defined as knowing that they are taking an anticoagulant as well as being aware of the need to check with the pharmacist or doctor before taking over the counter (OTC) medicines or herbal products. Key knowledge was reported by more patients who were prescribed a VKA and those who had an alert card, compared to those on a DOAC. Patients using an MCA had significantly less key knowledge than patients not using a MCA, and although fewer patients in care homes reported key knowledge, this was not found to be statistically significant (Table 2). More warfarin patients had a traditional alert card compared to DOAC patients, who were in possession of other types of alert cards or records (Table 3). Of all patients in possession of an alert card, three-quarters regarded alert cards as being useful or very useful (Fig. 2). Patients reported a preference for laminated or plastic cards as “these alert cards easily became tatty” and the perceived usefulness of alert bracelets or necklaces over alert cards in an emergency.

**Table 2:** Factors affecting patients’ key knowledge

	Key knowledge reported		*p* value (Chi squared test)
	Yes (n = 14274^a^)	Total (n = 16994^a^)	
Not care home resident	13,970 (84.0%)	16,610	*p* = 0.168
Care home resident	110 (79.7%)	138	
Direct-acting oral anticoagulant	6481 (78.7%)	8237	*p* < 0.001
Vitamin K antagonist	7793 (89.0%)	8757	
Not using multi-compartment compliance aid	13,437 (84.7%)	15,869	*p* < 0.001
Using a multi-compartment compliance aid	834 (74.1%)	1125	
No alert card	2868 (77.3%)	3708	*p* < 0.001
Possession of an alert card	11,406 (85.8%)	13,286	

^a^Residency status not know for 246 patients

**Table 3:** Comparison of key knowledge, awareness and use of alert cards reported by patients on direct-acting oral anticoagulants and warfarin

	Direct-acting oral anticoagulant (n = 8237)	Warfarin (n = 8696)	*p* value (Chi squared test)
Aware of type of medication	7842 (95.2%)	8541 (98.2%)	*p* < 0.001
Aware of checking about over the counter medication	6647 (80.7%)	7822 (89.9%)	*p* < 0.001
*Traditional alert card*			
Verbal confirmation of this card	2548 (30.9%)	3676 (42.3%)	*p* < 0.001
Card seen by pharmacy staff	1071 (13.0%)	2378 (27.3%)	
Not known/not reported	825 (10.0%)	470 (5.4%)	
No card or unaware of card	3793 (46.0%)	2172 (25.0%)	
Other alert card	473 (5.7%)	762 (8.8%)	*p* < 0.001
Manufacturer’s alert card	4910 (59.6%)	601 (6.9%)	
Not known/not reported	671 (8.1%)	1188 (13.7%)	
No card	2183 (26.5%)	6145 (70.7%)	
Yellow book	569 (6.9%)	5673 (65.2%)	*p* < 0.001
Other information	1320 (16.0%)	693 (8.0%)	
Not known/not reported	1221 (14.8%)	533 (6.1%)	
No book or other information	5127 (62.2%)	1797 (20.7%)

### Compliance with national guidance for managing warfarin

Patients on warfarin were included in the assessment of compliance with national guidance (Table 4). 82% had their INR tested within the past 12 weeks, in line with national guidance. Almost half (46.3%) of warfarin patients contacted were on alternate day dosing, which is contrary to the alert recommendations, and the dose instruction ‘as directed’ was used for 82.5% of warfarin patients. Good compliance (97.6%) was observed for exclusion of warfarin from MCA, with DOACs, notably edoxaban (21.7% patients on edoxaban), apixaban (20.9% of patients on apixaban) and dabigatran (9.5% of patients) more likely to be dispensed in MCA (Table 5).

**Table 4:** Monitoring and record keeping of patients prescribed warfarin

	All warfarin patients (n = 10,802)	Warfarin patients contacted (n = 8696)
INR record seen	3599 (33.3%)	3283 (37.8%)
*INR last tested*		
Less than 4 weeks ago	5971 (55.3%)	5477 (63.0%)
Between 4 and 12 weeks ago	2785 (25.8%)	2470 (28.4%)
More than 12 weeks ago	120 (1.1%)	102 (1.2%)
Not known	19,276 (17.8%)	644 (7.4%)
*INR value*		
Less than 2	645 (6.0%)	603 (6.9%)
Between 2 and 5	7535 (69.8%)	6908 (79.5%)
More than 5	57 (0.5%)	52 (0.6%)
Not known	2565 (23.7%)	1130 (13.0%)
INR recorded in pharmacy	3737 (34.6%)	3330 (38.3%)
*Required to halve or cut tablets for required dose*		
Yes	548 (5.1%)	496 (5.7%)
No	8716 (80.7%)	7698 (88.6%)
Not known	1538 (14.2%)	499 (5.7%)
*Reported alternate day dosing regimen*		
Yes	4387 (40.6%)	4027 (46.3%)
No	4592 (42.5%)	4013 (46.2%)
Not known	1823 (16.9%)	653 (7.5%)
*Patient aware dietary changes can affect anticoagulation*		
Yes	7881 (73.0%)	7061 (81.2%)
No	1251 (11.6%)	1140 (13.1%)
Not known	1670 (15.5%)	492 (5.7%)
Prescriber contacted due to safety concerns	210 (1.9%)	199 (2.3%)

*INR* international normalised ratio

**Table 5:** Anticoagulant dosing instructions and use of multi-compartment compliance aids

	Dosage specified		Multi-compartment compliance aid and type	
	Yes	Yes—dose not consistent	Multiple medicines per compartment	Single medicine per compartment
Acenocoumarol (n = 75)	12 (16.0%)	6 (8.0%)	3 (4%)	1 (1.3%)
Apixaban (n = 5493)	5424 (98.7%)	7 (0.1%)	996 (18.1%)	149 (2.7%)
Dabigatran (n = 728)	711 (97.7%)	2 (0.3%)	61 (8.4%)	15 (2.1%)
Edoxaban (n = 281)	276 (98.2%)	0	50 (17.8%)	11 (3.9%)
Phenindione (n = 2)	0	0	0	0
Rivaroxaban (n = 5243)	5179 (98.8%)	7 (0.1%)	731 (13.9%)	142 (2.7%)
Warfarin (n = 10,802)	944 (8.7%)	946 (8.8%)	186 (1.7%)	75 (0.7%)
Total (n = 22,624)	12,546 (55.5%)	968 (4.3%)	2420 (9%)	393 (1.7%)

### Pharmacist’s contributions

For patients on warfarin, the pharmacists contacted the prescriber in 210 cases, to address safety or medicines optimisation issues. Safety issues included recommendation to stop concomitant antiplatelet (1 patient) and DOAC (1 patient); patients reporting adverse drug events such as bleeding (13 patients), bruising (4 patients), others (7 patients); high INR requiring intervention (7 patients), out of range or missing INR values requiring re-measurement (4 patients); wrong dose taken by patient (5 patients); and drug: drug interactions (24 patients) requiring adjustment of treatments. Medicines optimisation issues included addressing inappropriate dispensing in MCA; provision of appropriate tablet strengths to ensure safe dose administration (14 patients); highlighting the need to plan for procedures or interventions (4 patients); identifying potential non-adherence (2 patients); and provision of anticoagulation information and record books (4 patients).

## Discussion

The majority of the patients had the knowledge and awareness of their anticoagulant treatment, especially those on warfarin and those with alert cards of some kind. This is similar to a recent report where patient awareness of the purpose of their anticoagulant therapy was found to be high [20] even though the level of patient knowledge surrounding their anticoagulant treatment has generally been reported as requiring improvement in the wider literature [20–22]. Due to the popularity and long-term establishment of VKAs, patient understanding has more commonly been assessed in VKA patients compared to DOAC patients [6, 22, 23]. More research has since been conducted to assess patient knowledge in both VKA and DOAC patients, as well as in DOAC patients only [24].

Although the evaluation found reasonable compliance with national patient safety alert recommendations for monitoring warfarin with most patients confirming that regular blood tests were in place, the INR results were not always known by the dispensing pharmacist. Dosage instructions were not specified for acenocoumarol and warfarin in the majority of cases. The latter may be reflective of the fact that warfarin dosing instructions vary depending on INR results and are therefore recorded in specific oral anticoagulant therapy books or similar records alongside the results. Relying on patient-held information to confirm safety in this way has repeatedly fallen short; there is clear need to rapidly operationalise a system which allows both prescribers and dispensers direct access to this information. Potential solutions are for these results to be included in the national Summary Care Record [25] or available on the NHS App [26].

Community pharmacists are in a key position to provide high-quality face-to-face care promoting the safe and effective use of anticoagulants and providing and reviewing patient-held booklets. In the current study, patients on DOACs and those whose medicines were dispensed in MCA were slightly less knowledgeable about their treatment and the need to inform other healthcare professionals before self-medicating to minimise risk of interactions and harm, whereas others have shown no difference in the overall anticoagulant knowledge in VKA and DOAC patients [20]. This may partly be a reflection of pharmacists self-confidence and knowledge providing care to patients on warfarin compared to DOACs [27, 28]. It may also relate to the variation in the types of alert cards and information booklets used despite national initiatives to standardise patient held anticoagulant information. Traditional yellow alert cards that included summary information to aid communication with other health professionals were more likely to be issued to patients on warfarin, whereas patients on DOACs were issued with a mix of medicines’ manufacturers and other regional or organisational alert cards [29, 30]. There is a good case for sharing the latter materials to avoid duplication of effort and variation as individual organisations seek to meet the information needs of DOAC patients. The findings also highlighted that patients had suggestions for the design and development of alert materials to facilitate better use and improve usefulness, which could be harnessed in a co-design approach for any future revised national materials.

A number of safety contributions were made by community pharmacists through identification of adverse reactions, out of range INR values, and concomitant prescribing of interacting medicines. Medicines optimisation issues relating to ease of administration and adherence issues were also identified. In particular, the results showed that ten percent of patients on dabigatran had this dispensed in an MCA, which is against the manufacturer’s recommendation to store in original packaging to protect from stability issues [31] and a potential safety concern.

The evaluation has a number of limitations. Participation was voluntary and related to a contractual requirement under NHS regulation, which may have led to a self-selection bias. However, there was good geographical distribution across the country with more than 3000 patients reported from each region and participation from 13% of all pharmacies in England 2017–2018 [32]. It was also the third centrally co-ordinated medicines safety collaborative audit and evaluation of this type and had the largest sample of both pharmacies and patients [9, 10]. Inclusion to the survey was dependent on patients having anticoagulants dispensed through the participating pharmacists. The survey results may be limited due to a small proportion of prescriptions not being collected by the patient themselves, and instead, being delivered or collected by the patients’ representative, leading to missing or potentially incomplete or inaccurate information. This was a point prevalence survey of patients’ anticoagulation and may not reflect longitudinal issues. A number of patients were reported as being on multiple anticoagulants. However, the evaluation was not designed to capture the potential reasons for this, which may include a transition period from one anticoagulant to another, or prescribing errors and unintended duplicate therapy.

Overall, this service evaluation highlighted a number of key aspects to improve anticoagulant safety. There is a need to update and standardise the type and format of alert cards, to improve usefulness and use by patients. Community pharmacists require a supportive infrastructure to work in partnership with prescribers and patients. Additionally, this evaluation demonstrated that collaborative working across national pharmacy bodies can drive engagement with individual community pharmacies to support national medicines safety priorities. There is significant opportunity for such collaboratives to make an important contribution to the WHO goal of halving severe avoidable medication related harm by 2022.

## Conclusion

Anticoagulants are the class of medicines most frequently associated with serious patient harm. Overall, patients demonstrated a good awareness and knowledge about their anticoagulation treatment, concomitant medicines use and monitoring, but did not always use or value alert cards. Community pharmacists have a role in improving the safety of anticoagulation treatment and patient knowledge, through regular reviews and education.

## Availability of data and materials

On request from authors.
